# Antibiotic resistance, biofilm formation, and virulence genes of *Streptococcus agalactiae* serotypes of Indian origin

**DOI:** 10.1186/s12866-023-02877-y

**Published:** 2023-07-05

**Authors:** Shalini Verma, Monika Kumari, Anurag Pathak, Vikas Yadav, Atul Kumar Johri, Puja Yadav

**Affiliations:** 1 Department of Microbiology, Central University of Haryana, Mahendergarh, Haryana India; 2 Department of Statistics, Central University of Haryana, Mahendergarh, Haryana India; 3 School of Life Sciences, Jawaharlal Nehru University, New Delhi, 110067 India

**Keywords:** Biofilm, Antibiotic resistance, Group B *Streptococcus*, Immunocompromised patients, Virulence factors

## Abstract

**Background:**

Group B *Streptococcus* (GBS) is a causative agent of various infections in newborns, immunocompromised (especially diabetic) non-pregnant adults, and pregnant women. Antibiotic resistance profiling can provide insights into the use of antibiotic prophylaxis against potential GBS infections. Virulence factors are responsible for host–bacteria interactions, pathogenesis, and biofilm development strategies. The aim of this study was to determine the biofilm formation capacity, presence of virulence genes, and antibiotic susceptibility patterns of clinical GBS isolates.

**Results:**

The resistance rate was highest for penicillin (27%; *n* = 8 strains) among all the tested antibiotics, which indicates the emergence of penicillin resistance among GBS strains. The susceptibility rate was highest for ofloxacin (93%; *n* = 28), followed by azithromycin (90%; *n* = 27). Most GBS strains (70%; *n* = 21) were strong biofilm producers and the rest (30%; *n* = 9) were moderate biofilm producers. The most common virulence genes were *cylE* (97%), *pavA* (97%), *cfb* (93%), and *lmb* (90%). There was a negative association between having a strong biofilm formation phenotype and penicillin susceptibility, according to Spearman’s rank correlation analysis.

**Conclusion:**

About a third of GBS strains exhibited penicillin resistance and there was a negative association between having a strong biofilm formation phenotype and penicillin susceptibility. Further, both the strong and moderate biofilm producers carried most of the virulence genes tested for, and the strong biofilm formation phenotype was not associated with the presence of any virulence genes.

**Supplementary Information:**

The online version contains supplementary material available at 10.1186/s12866-023-02877-y.

## Introduction

*Streptococcus agalactiae*, also known as Group B *Streptococcus* (GBS), is a Gram-positive opportunistic pathogen that causes severe infections such as sepsis, pneumonia, and meningitis in neonates [1, 2]. Based on their capsular polysaccharide composition, ten serotypes of GBS have been reported (Ia, Ib, II, III, IV, V, VI, VII, VIII, and IX). Among the serotypes, Ia, Ib, II, III, and V are the most common [3–5]. In India, serotypes Ia and III were found to be the most common serotypes [6]. GBS can form aggregates on interfaces, known as biofilms, which facilitate GBS persistence under environmental stresses and allow GBS to survive in hostile environments and protect against antibiotics [7–9]. Approximately 65% of infections are associated with biofilms and they have a significant role in many persistent infections [10]. Notably, low doses of certain antibiotics induce biofilm formation, indicating that biofilm development may be involved in the global response to external stresses, including antibiotics [11]. In recent years, the ability of GBS to form biofilms has gained significant attention due to its possible role in survival and pathogenesis [12]. The GBS biofilm formation ability was reported to be higher in asymptomatic compared to symptomatic pregnant women [13]. Importantly, biofilm formation by GBS depends on the expression of various virulence factors [14].

Upon streptococcal infection, virulence factors help the bacteria to adapt to changing host environments and provide survival strategies, including biofilm formation, that facilitate the disease manifestation [9, 15]. In this regard, GBS expresses a diverse array of surface-associated and secreted virulence factors that mediate specific interactions with host cells and interfere with innate immune clearance mechanisms. Some of the virulence factors have been identified and characterized, including adhesion and invasion factors that assist the bacteria in colonizing both epithelial and endothelial tissues and crossing these host barriers. GBS surface proteins called adhesins enable the bacteria to make persistent and intimate contacts with the host cells [16]. Additionally, evasion factors can decrease neutrophil recruitment and prevent complement cascade binding, pore-forming toxins can damage host cells, and other factors can repel or otherwise induce resistance to antimicrobial peptides [17].

The nature of GBS infections may be determined by various virulence genes such as *gbs67* (encodes an ancillary protein of pili and promotes adherence and invasion)*, cylE* (encodes β-hemolysin, which promotes invasion of host cells)*, cfb* (encodes Christie–Atkins–Munch–Peterson [CAMP] factor and forms pores in host cell membranes)*, scpB* (encodes the surface enzyme scpB [C5a peptidase], which promotes adherence and prevents neutrophils from reaching the infection site)*, lmb* (encodes laminin-binding protein and promotes adherence to host laminin), and *pavA* (encodes an aggregation factor and promotes binding to immobilized fibronectin) [18, 19].

To the best of our knowledge, no information is available on the role of biofilm formation and antibiotic resistance in GBS strains of Indian origin. In the current study, we assessed the biofilm formation by 30 clinical GBS isolates and found a correlation between strong biofilm formation capacity and penicillin resistance. We also analyzed the distribution pattern of various virulence genes to determine the associations between biofilm formation capacity and virulence genes, but we found no associations.

## Methods

### GBS serotypes and culture conditions

A total of 250 samples (urine, semen, etc.) that were previously isolated from men and women (aged 10–83 years) at 12 hospitals in Delhi, Gurgaon, and Jaipur, India, 30 samples were identified as containing GBS by latex agglutination tests using a Streptex GBS typing antisera kit [6]. This was confirmed by PCR targeting the GBS-specific *atr* gene, which encodes an amino acid transporter gs0538): 5'-CAA CGA TTC TCT CAG CTT TGT TAA-3' and 5'-TAA GAA ATC TCT TGT GCG GAT TTC-3', producing a 780-bp fragment [20]. These 30 GBS strains were inoculated in Todd Hewitt Broth medium (THB; Himedia Laboratories, India). Briefly, each culture from frozen stock was streaked using a sterile loop on a 5% sheep blood agar plate and incubated at 37 °C for 24–30 h. Serotyping was conducted using latex agglutination tests, utilizing a GBS typing antisera kit (Denka Seiken Kit, Japan) [6].

### Antibiotic susceptibility tests

To determine the antibiotic resistance profile of the 30 GBS strains, they were tested against six antibiotics, i.e., penicillin (10 units), clindamycin (2 µg), erythromycin (15 µg), gentamicin (10 µg), ofloxacin (5 µg), and azithromycin (15 µg), using the Kirby–Bauer disk-diffusion method according to the Clinical and Laboratory Standards Institute (CLSI) guidelines [21]. The six antibiotics were selected from various antibiotic classes (which exhibit different mechanisms of bacterial killing), as these antibiotics are the most widely used for the treatment of GBS infections. Penicillin or ampicillin are used as first-line therapy for intrapartum antibiotic prophylaxis, while clindamycin is used for penicillin-allergic patients [22].

Briefly, homogeneous GBS suspensions of 0.5 McFarland turbidity standards were prepared from fresh bacterial cultures, as described previously [23]. This was done by suspending GBS colonies in 5 ml physiological saline and adjusting the turbidity to 0.5 McFarland turbidity standards [19], which was used as a reference and has an optical density comparable to 1.5 × 10^8^ bacterial colony-forming units (CFU/ml). Each GBS suspension was used to form a bacterial lawn on Mueller–Hinton agar with 5% sheep blood. Specific antibiotic discs were placed onto the lawn using sterile forceps and were incubated overnight at 37 °C. The zone of inhibition was manually measured and the results were interpreted as susceptible, intermediate, or resistant according to the CLSI guidelines [21].

### Biofilm formation assays

Biofilm formation was assessed by Congo red agar (CRA) assay (qualitative assay) and crystal violet assay (CVA; quantitative assay).

For the CRA assay, 0.08% CRA plates containing THB supplemented with 1% glucose were inoculated with GBS suspension and incubated at 37 °C for 24 h. Thereafter, the results were interpreted according to the colony phenotypes and changes in color. The formation of black colonies with slime production was used as an indicator for biofilm formation [24, 25]. For the CVA, a colony of each GBS strain cultured on blood agar plates was used to inoculate 5 ml THB supplemented with 1% glucose. After incubation at 37 °C until the optical density at 600 nm (OD_600_) reached ~ 0.5, 100μL culture was added to a 96-well microtiter plate along with 100μL fresh THB with 1% glucose. After incubation at 37 °C for 48 h under static conditions, the plate was gently washed three times with 1X phosphate-buffered saline (PBS; pH 7.4) followed by heat fixation at 60 °C for 1 h and staining with 100μL of 0.5% CV for 5 min. Next, the plate was washed three times with 1X PBS, the remaining CV was solubilized by adding 200μL of 95% ethanol, and the mixture was incubated at room temperature for 10 min. The OD_595_ of each well was measured using an enzyme-linked immunosorbent assay (ELISA) plate reader (BioTek Synergy™ H1 hybrid multi-mode microplate reader, USA). OD_595_ < 2, 2–4, 4–8, and > 8 were considered to indicate non-biofilm producers, weak biofilm producers, moderate biofilm producers, and strong biofilm producers, respectively [26]. *Pseudomonas aeruginosa* (MTCC 2297) and THB + 1% glucose (medium only) were used as the positive and negative controls, respectively [27, 28]. All experiments were performed in triplicate.

### Detection of virulence genes by PCR

Six virulence genes (*gbs67, cylE, cfb, scpB, lmb,* and *pavA*) were detected by conventional PCR using gene-specific primers (Supplementary Table S1), due to their roles in biofilm formation.

First, genomic DNA was isolated from the GBS strains using the cetyltrimethylammonium bromide method (CTAB) [29]. In brief, 50 ml of log-phase GBS culture (OD_600_ of 0.5) in THB was centrifuged at 6000 rpm for 5 min at room temperature. The pellet was washed twice with 5 ml of 0.1 M Tris buffer (pH 8.0). Next, 50 µl of lysozyme (100 mg/ml), 200 µl of 10% sodium dodecyl sulfate, and 60 µl of proteinase K (60 mg/ml) were added and incubated at 65 °C for 2 h. Further, 500 µl of 5 M NaCl and 800 µl of 10% CTAB were added, mixed gently, and incubated for 30 min at 65 °C. Thereafter, 15 ml of chloroform: isoamyl alcohol (24:1) was added and the mixture was vortexed for 10 s and centrifuged at 12,000 rpm for 10 min. The upper aqueous phase was collected and an equal volume of phenol: chloroform (1:1) was added to it. After vortexing for 5 s and centrifugation at 12,000 rpm for 5 min, the upper aqueous phase was collected and 2 µl of RNAse (10 mg/ml) was added. After incubation at 37 °C for 30 min, an equal volume of chilled absolute ethanol was added and the mixture was incubated for 2 h at -20 °C and then centrifuged at 12,000 rpm for 10 min. The DNA pellet was washed with chilled 70% ethanol (1 ml) and centrifuged at 12,000 rpm at 4 °C for 15 min. The DNA pellet was dissolved in nuclease-free water. Quantification and purity of the extracted genomic DNA was checked by measuring the absorbance at 260 and 280 nm on UV spectrophotometer.

The PCR mixture contained 2 mM MgCl_2_, 5 pmol of each forward and reverse primer, 0.5 mM dNTPs mix, 100 ng genomic DNA template, and 2 units high-fidelity DNA polymerase. The PCR conditions used were as follows: initial denaturation at 95^◦^C for 5 min, 30 cycles of 95^◦^C for 45 s, 40–57^◦^C (depending on primer melting temperature) for 45 s, and extension at 72^◦^C for 1 min, followed by a final extension at 72^◦^C for 5 min. The PCR products were resolved on 1.2% Tris–acetate–EDTA (TAE) agarose gel and visualized under UV light. The gyrase subunit A (*gyrA*) gene was used as an internal standard [30].

### Statistical analysis

Differences in the mean zone of inhibition among the six antibiotics were assessed by analysis of variance (ANOVA) followed by Tukey's post hoc test. Chi-square test was used to assess the associations of virulence genes (presence or absence) with biofilm formation capacity (moderate or strong [no weak biofilm producers or non-biofilm producers were found]) among the GBS strains, because the variables are not continuous [31]. Spearman's rank correlation analysis was used to assess the correlations between antibiotic resistance (X) and biofilm formation capacity (Y) among the GBS strains. Here, association refers to the general relationship between categorical/variables, whereas Pearson correlation coefficient refers to a linear relationship between two quantitative variables. R software was used to analyze the data and construct graphs, and *p* < 0.05 was considered statistically significant in all analyses.

## Results

### Antibiotic susceptibility

**Fig. 1:**
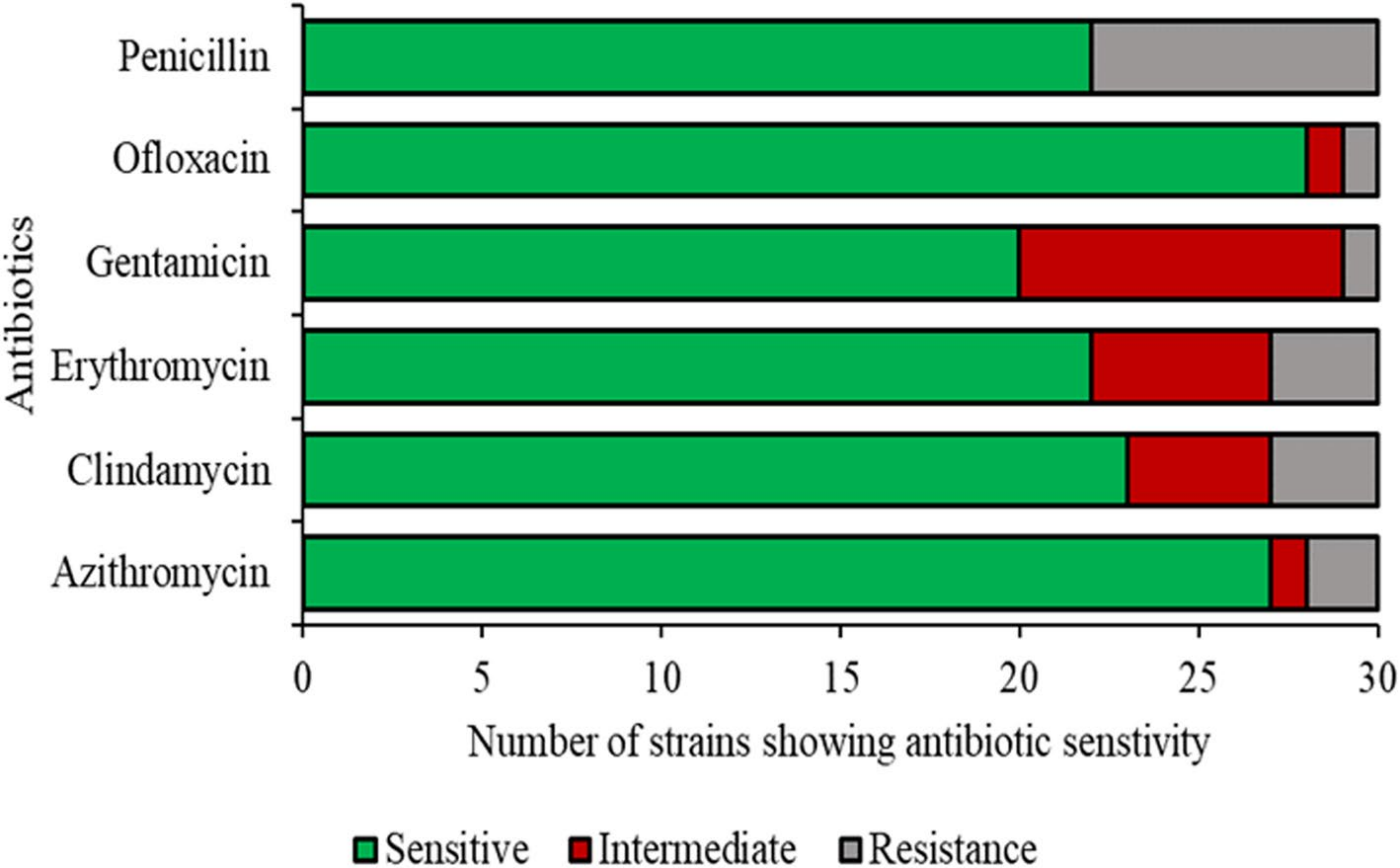
Resistance of 30 GBS strains to six different antibiotics

Among the tested antibiotics, penicillin had the highest resistance rate among the GBS strains, i.e., 27% (*n* = 8). The intermediate and susceptibility rates were 0% (*n* = 0) and 73% (*n* = 22), respectively. The resistance, intermediate, and susceptibility rates were 10% (*n* = 3), 17% (*n* = 5), and 73% (*n* = 22), respectively, for erythromycin, 10% (*n* = 3), 13% (*n* = 4), and 77% (*n* = 23), respectively, for clindamycin, and 7% (*n* = 2), 3% (*n* = 1), and 90% (*n* = 27), respectively, for azithromycin. The resistance rates for gentamicin and ofloxacin were similar i.e., 3% (*n* = 1), but the intermediate rate, i.e., 30% (*n* = 9) and 3% (*n* = 1), and susceptibility rate, i.e., 67% (*n* = 20) and 93% (*n* = 28), were different (Fig. 1, Supplementary Table S2). The results show that 33% (*n* = 10) of GBS strains were susceptible to all antibiotics while the remaining 67% (*n* = 20) were either resistant or intermediate to at least one antibiotic. 37% (*n* = 11) were resistant to at least one antibiotic.

To determine whether the six antibiotics affect the GBS strains to the same degree or differently (in terms of their inhibition on bacterial growth), we statistically compared the mean zone of inhibition among the six antibiotics (Supplementary Table S2). The difference in the mean zone of inhibition between penicillin and gentamicin was high i.e., 7.9 mm, which indicates that they were not similarly effective. Interestingly, the difference between azithromycin and erythromycin was only 0.05 mm, which suggests that they had a similar inhibitory effect on GBS strains (Figure S1, Supplementary Table S2).

We also revealed associations between the GBS serotypes and antibiotic resistance. One isolate (3%) (serotype V, G28) exhibited MDR to three classes of antibiotics (clindamycin, erythromycin and penicillin). Serotype III strains had the highest resistance levels, i.e., 50% were resistant to azithromycin, erythromycin, and penicillin, and 25% were resistant to both clindamycin and ofloxacin (Figure S2). Regarding serotype V strains, 100% were resistant to penicillin, and 50% were resistant to erythromycin and clindamycin. Regarding serotype Ia strains, 43% were resistant to penicillin and 14% were resistant to clindamycin (Figure S2).

### Detection of biofilm-producing phenotype

**Fig. 2:**
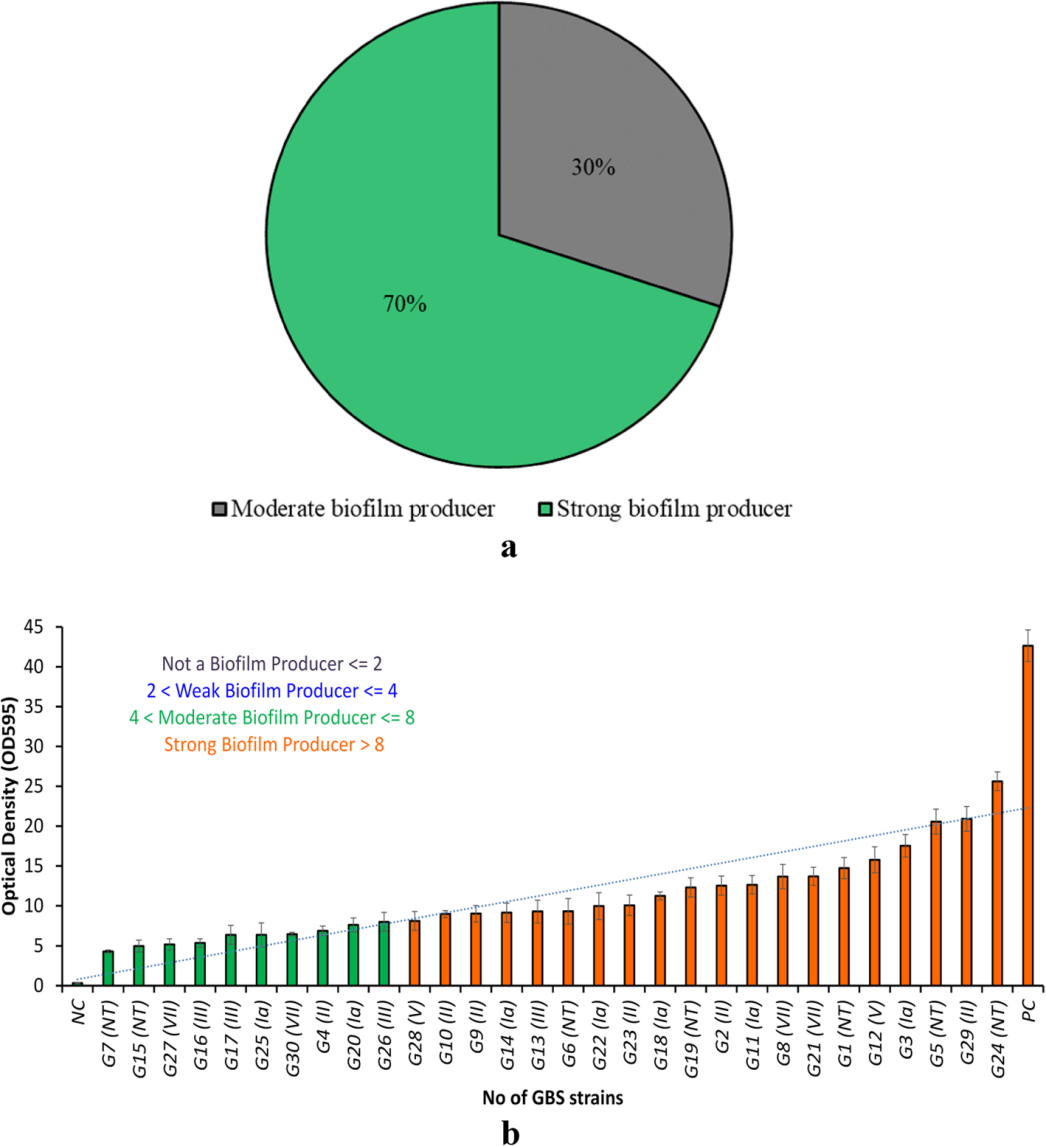
**a** Percentages of strong and moderate biofilm producers among the 30 GBS strains. **b** Biofilm formation of GBS strains based on crystal violet assays (CVA). Optical density at 595 nm indicates biofilm formation capacity. Of the 30 GBS strains, the G24 (NT) strain had the strongest biofilm formation capacity, whereas the G7 strain (NT) had the weakest (but still moderate) biofilm formation capacity. PC: positive control (*Pseudomonas aeruginosa)* [27]. NC: negative control (THB + 1% glucose medium) [28]

CRA was used to qualitatively assess biofilm-producing phenotypes among the GBS strains, based on extracellular polymeric substances (EPS) production. A change in the color of the media from red to black was used to designate GBS as a biofilm producer. Based on the CRA assay, all GBS strains (100%) produced biofilm**.** CVA was used to quantitatively assess biofilm-producing phenotypes among the GBS strains. 70% (*n* = 21) and 30% (*n* = 9) of GBS strains were strong and moderate biofilm producers, respectively (Fig. 2a). Interestingly, no weak biofilm producers or non-biofilm producers were found. The biofilm formation capacity of each isolate is summarized in Fig. 2b and Supplementary Table S3.

**Fig. 3:**
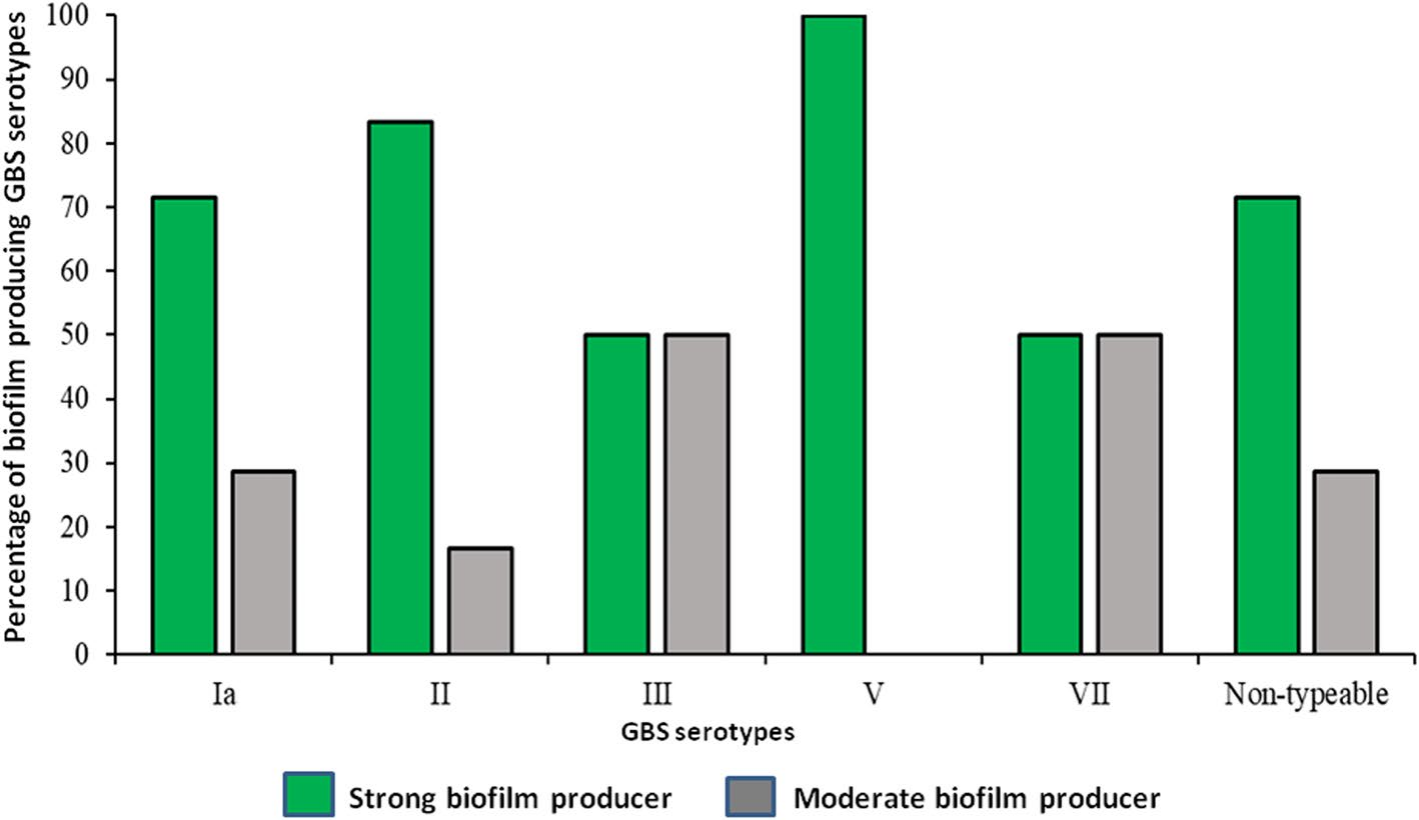
Biofilm formation capacity of GBS serotypes. Biofilm formation capacity was assessed by Congo red assays (CRA) and crystal violet assays (CVA)

Comparative growth rate analyses showed that there was no significant difference in the growth rate between the strong and moderate biofilm producers (Figure S3). Serotypes Ia, and V have higher number of strains producing strong biofilm compared to serotypes III and VII (Fig. 3). Invasive serotypes were strong biofilm producers compared to the colonizing serotypes which were moderate biofilm producers (Figure S4).

### Correlations between biofilm formation and antibiotic resistance

**Fig. 4:**
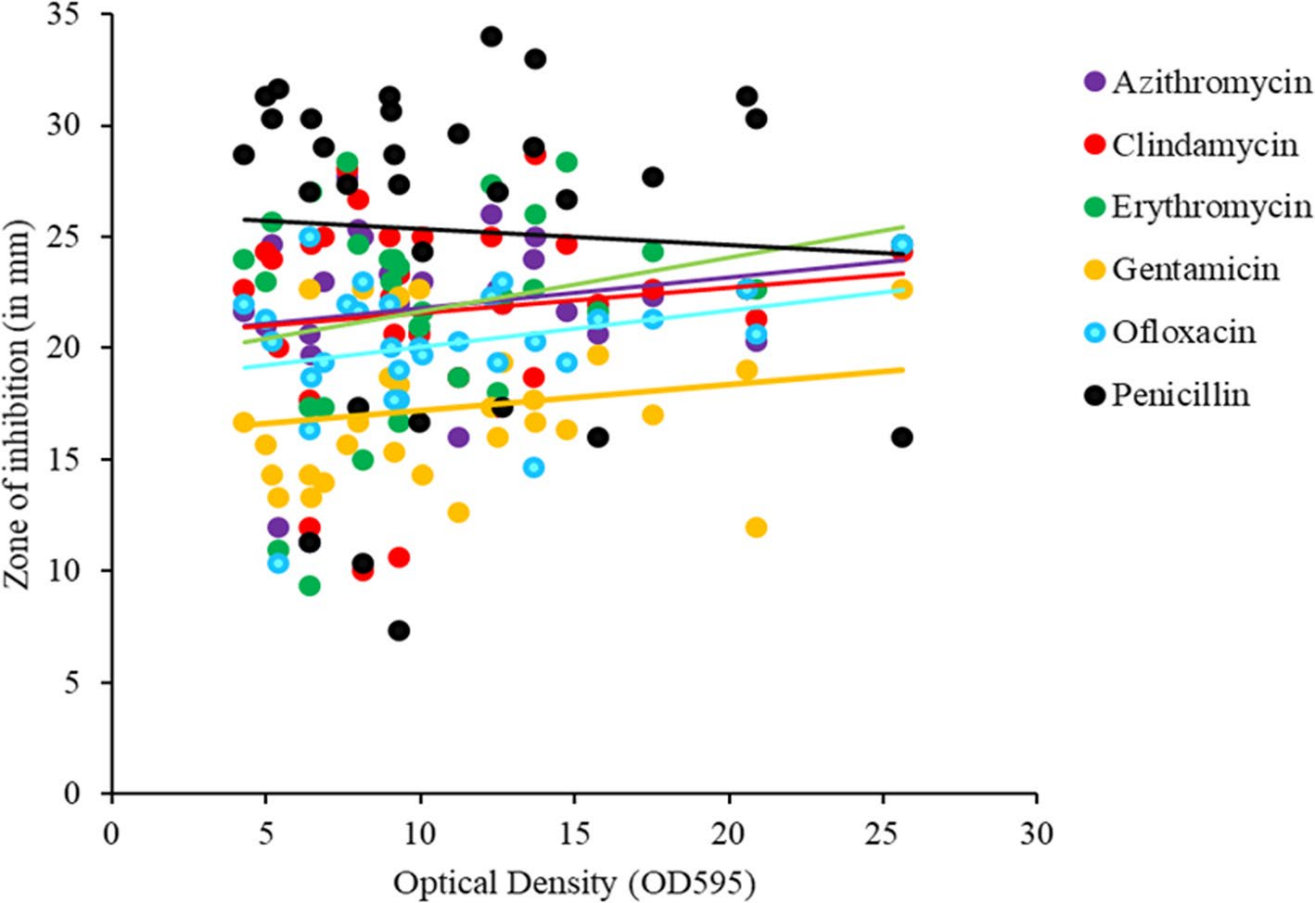
Correlation of antibiotic susceptibility with biofilm formation capacity in GBS strains

Spearman's rank correlation coefficient (r_s_) was used to assess the correlations between biofilm formation (X) and antibiotic susceptibility (Y) [28]. There were non-significant positive correlations between biofilm formation and susceptibility to azithromycin (r_s_ = 0.2, *p* > 0.05), clindamycin (r_s_ = 0.126, *p* > 0.05), erythromycin (r_s_ = 0.269, *p* > 0.05), gentamicin (r_s_ = 0.188, *p* > 0.05), and ofloxacin (r_s_ = 0.289, *p* > 0.05). However, for penicillin, there was a significant negative correlation (r_s_ = -0.051, *p* < 0.05), which suggests that the GBS strains that had increased biofilm formation capacity were resistant to penicillin (Fig. 4, Supplementary Table S2). When biofilm increases, the effect of penicillin decreases, i.e., strong biofilm-forming strains were more likely to be resistant.

### Detection of virulence genes and their associations with biofilm formation

**Fig. 5:**
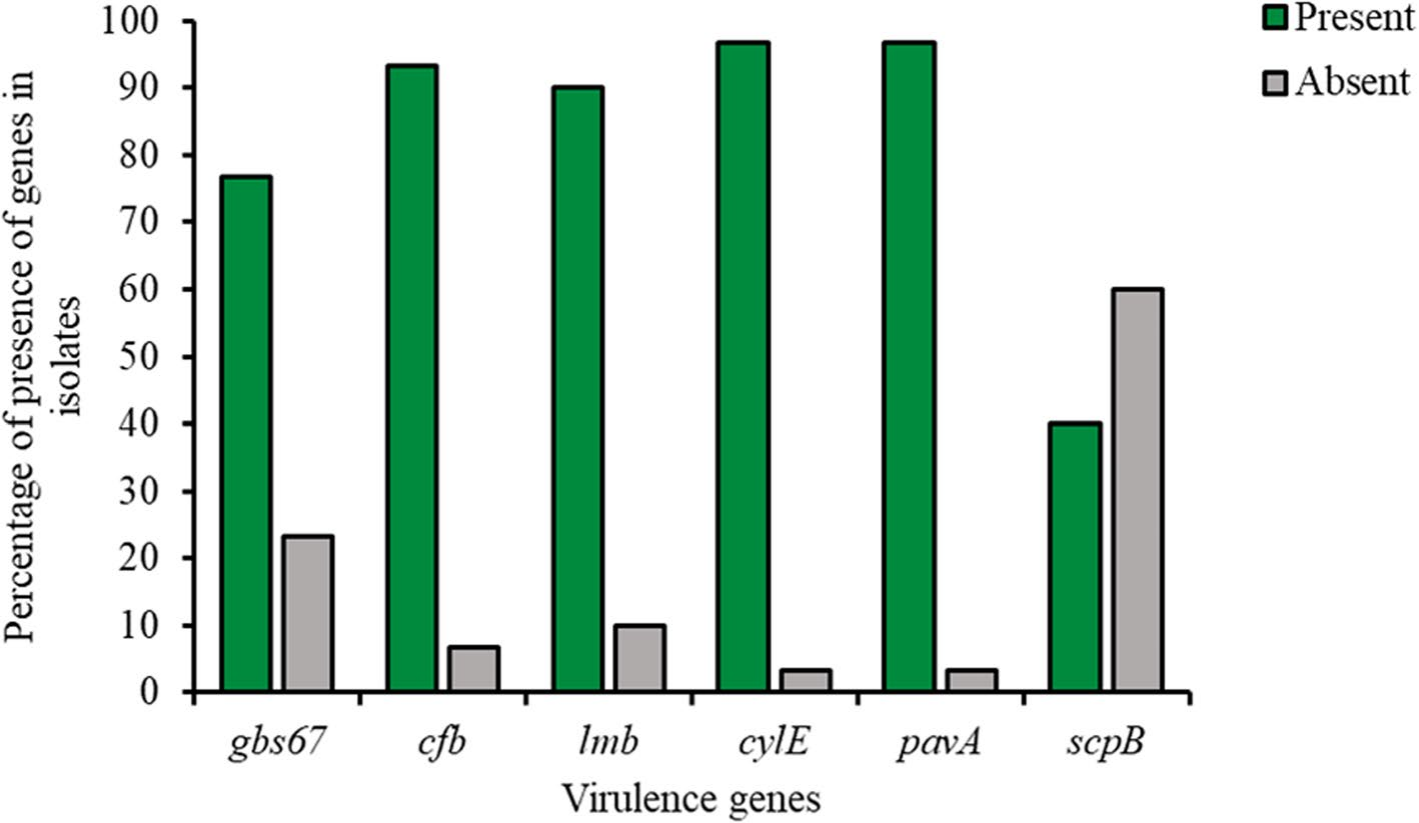
Distribution of virulence genes in GBS strains

**Fig. 6:**
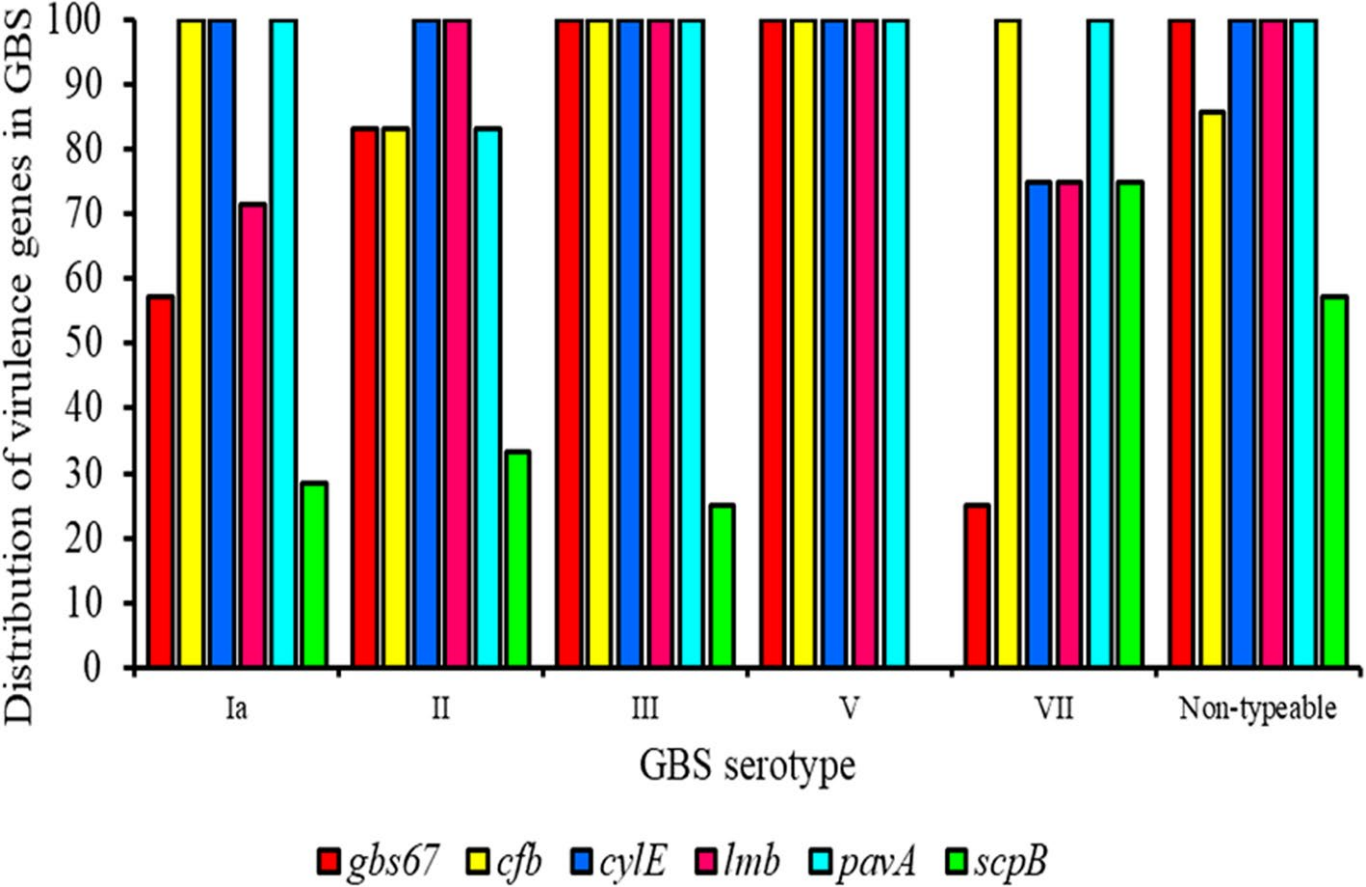
Distribution of virulence genes in GBS serotypes

The virulence genes identified were *cylE* (97%), *pavA* (97%), *cfb* (93%), *lmb* (90%), *gbs67* (77%), and *scpB* (40%) (Fig. 5, Supplementary Table S3). Except for *scpB*, there were no significant differences in virulence genes between serotypes III and V (Fig. 6).

**Fig. 7:**
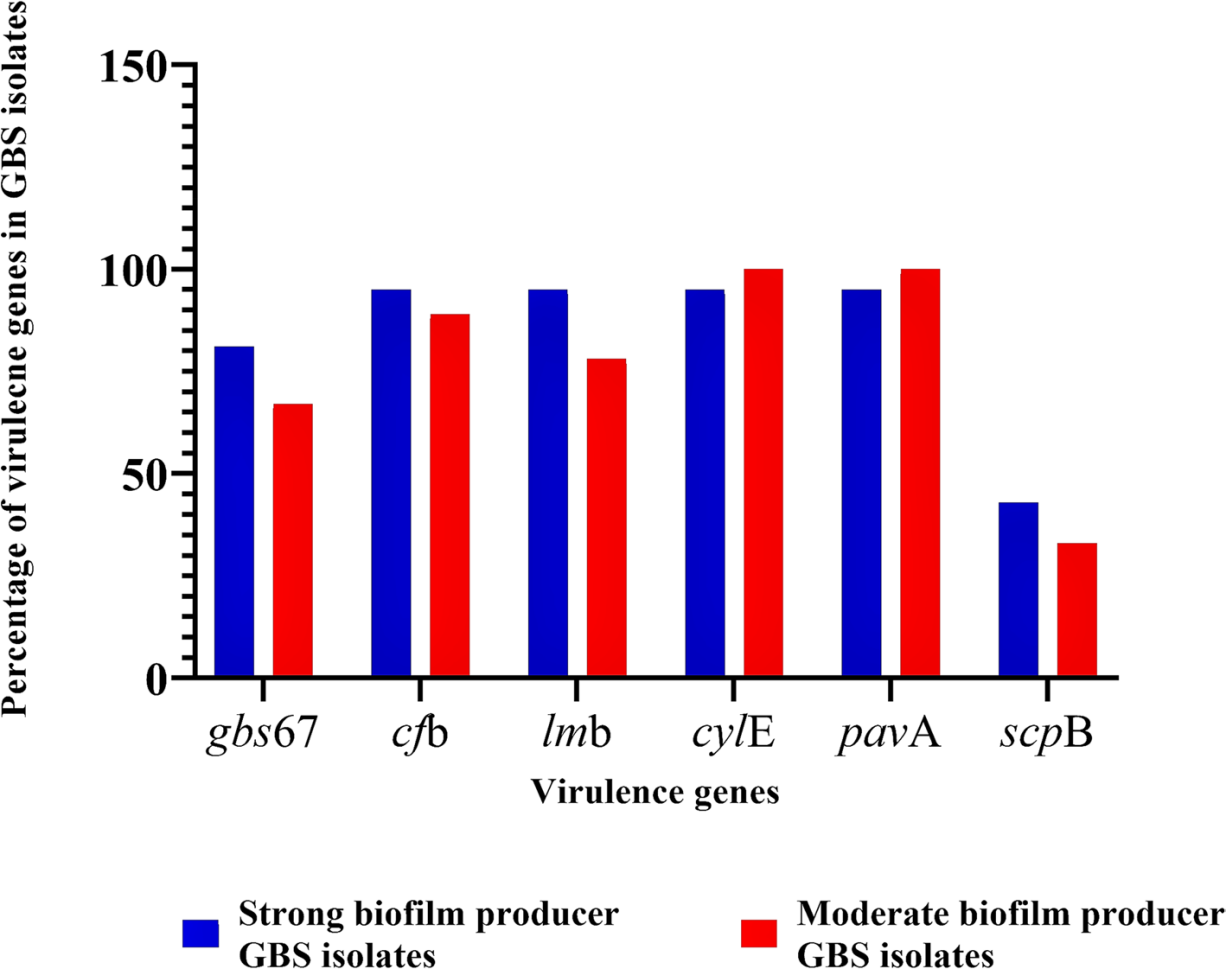
Distribution of virulence genes in strong and moderate biofilm-producing GBS strains

Most strains had multiple virulence genes, highlighting GBS strains’ ability to adhere, colonize, and invade host tissues. Regarding the strong biofilm producers, *cfb*, *lmb*, *cylE,* and *pavA* were present in 95%, followed by *gbs67* (81%) and *scpB* (43%). Regarding the moderate biofilm producers, *cylE* and *pavA* were present in all of them (100%), followed by *cfb* (89%), *lmb* (78%), *gbs67* (67%), and *scpB* (33%). Clearly, a low occurrence of *scpB* was observed in both strong and moderate biofilm producers (Fig. 7).

Further, chi-square test indicated that there were no significant associations between the occurrence of virulence genes (presence or absence) and biofilm formation capacity (strong or moderate [no weak biofilm producers or non-biofilm producers were found]) (*p* > 0.05).

## Discussion

Extensive use and misuse of antibiotics has resulted in the emergence of antibiotic resistance in bacterial species, which is a global concern [32]. In recent years, the emergence of antibiotic resistance among clinical GBS isolates has emphasized the need for continuous monitoring of antibiotic resistance patterns. In the present study, antibiotic resistance patterns in GBS clinical isolates from Delhi, Gurgaon, and Jaipur, India, were evaluated.

We found that out of the 30 GBS strains, 33% (*n* = 10) were susceptible to all six antibiotics tested and the remaining 67% (*n* = 20) were resistant/intermediate to at least one antibiotic. 37% (*n* = 11) and 43% (*n* = 13) were resistant or intermediate, respectively, to at least one antibiotic. However, no strains were resistant to all six antibiotics (azithromycin, erythromycin, clindamycin, gentamicin, ofloxacin, and penicillin). However, in the present study we have used only six antibiotics to assess the antibiotic susceptibility pattern of GBS isolates. Further, it would be interesting to check the antibiotic susceptibility of these GBS isolates against vancomycin which is being used against penicillin resistant GBS isolates and linezolid which has shown antibacterial activity against drug resistant GBS isolates.

MDR in bacteria occurs by the accumulation, on resistance (R) plasmids or transposons, of multiple genes that each encode resistance to a specific agent and/or by the action of multidrug efflux pumps, each of which can pump out more than one drug type. In our GBS strains, these genes were absent, not expressed, did not accumulate together, or were in the beginning stages of developing full resistance to the tested antibiotics. Hence, none of the GBS strains had resistance against all six antibiotics tested. As we did not explore the genetic causes of resistance to antibiotics or classes of antibiotics, which can vary within a species, this needs further investigation [33].

A previous study in Vietnam reported that, among GBS strains isolated from pregnant women, resistance was highest for tetracycline (89.66%), followed by erythromycin (76.23%) and clindamycin (58.21%) [34]. In Chinese neonates, the erythromycin resistance rate was much higher (92.5%) than in Taiwan (58.3%) and Western countries (11.5 to 32%) [35–38]. In our study, clindamycin (10%) and erythromycin (10%) resistance rates in India were both relatively high compared to resistance to other antibiotics except penicillin. In GBS strains, increased erythromycin resistance has been reported to be primarily caused by ribosomal modification by methyl-transferases encoded by erythromycin ribosomal methylase *(erm)* genes (*ermB, erm A/TR*) or by macrolide efflux pumps encoded by the *mefA/E* genes, which provide cross-resistance to macrolide, lincosamide, and streptogramin B (MLS_B_) antibiotics. The less frequent clindamycin resistance in GBS strains has been reported to be caused by changes to ribosomal translocation, which are related to the *linB* genes [39–41]. We speculate that similar mechanisms may exist in Indian GBS strains, hence they have developed clindamycin and erythromycin resistance, but this explanation needs further investigation.

GBS is considered to be susceptible to beta-lactam antibiotics and some countries use intrapartum prophylaxis regimes to prevent GBS infections. Reduced susceptibility to beta-lactams (including penicillin) has been observed among GBS strains [42–45]. We found that 27% of our GBS isolates exhibited penicillin resistance and serotypes III and V demonstrated increased penicillin resistance, which indicates the emergence of penicillin-resistant GBS strains in the Delhi National Capital Region. Penicillin resistance has also been reported in other parts of the world [46–48]. In Zimbabwe, a penicillin resistance rate of 69.8% was reported [19], and in Ethiopia, the rate was 77.3% [49], which supports our data showing a relatively high rate of penicillin resistance (27%) in the Delhi National Capital Region. Increased penicillin resistance among GBS isolates has been reported to be due to mutations in penicillin-binding proteins, which affect the binding capacity of penicillin [50, 51]. In a study conducted by Metcalf et al., [51], whole-genome sequencing was used to predict penicillin resistance in GBS strains from the USA; penicillin-resistant GBS strains were found to have mutation in *pbp2x* and other penicillin-binding protein genes, which contributed to reduced susceptibility to penicillin [51]. In a detailed study by Kitamura et al., the rates of cefotaxime (28%), ceftriaxone (36%), and levofloxacin (93%) resistance were high among GBS with reduced penicillin susceptibility [50]. We hypothesize that a similar mechanism, involving penicillin-binding proteins, may exist in the case of GBS strains of Indian origin. As we did not investigate this, it needs further investigation.

Overuse of antibiotics not only contributes to the emergence of biofilm-mediated MDR strains, but also helps microbes to adapt to many external stress conditions by forming biofilms. It has been suggested that biofilm formation plays an important role in antibiotic resistance by decreasing the antibiotic penetration rate and regulating bacterial gene expression, which makes it hard to treat bacterial infections [52, 53]. Bacteria in biofilms can be resistant to the immune system, antibiotics, and other treatments [54]. Similar to other streptococci, GBS can form biofilm-like three-dimensional structures that facilitate colonization and survival in the host. In the current study, we assessed the biofilm production of clinical GBS isolates and observed that all of them were able to form biofilms. Most of the GBS isolates were found to be strong biofilm producers and the rest were moderate biofilm producers. There was no significant difference in the growth rate between the strong and moderate biofilm producing GBS serotypes, which indicates that the difference in biofilm formation was not due to the growth rate. It was previously reported that antibiotic resistance can be achieved by biofilm production and both factors have an association with each other [28]. In uropathogenic *E. coli* isolates, biofilm-forming isolates were more associated with the MDR phenotype compared to the non-biofilm producers [55]. To determine whether there was a relationship between antibiotic susceptibility and biofilm formation capacity among our GBS isolates, we used Spearman's rank correlation analysis. Penicillin susceptibility was the only type of antibiotic susceptibility that exhibited a correlation with biofilm formation capacity, with a negative correlation being observed. However, for other tested antibiotics, no such relationship was observed. The GBS strains that were strong biofilm producers were not susceptible to penicillin; the effect of penicillin decreased in these strains, potentially due to reduced penicillin penetration of the strong biofilm.

Further, it has been reported that virulence genes are involved in pathogenicity by enhancing biofilm formation, which suggests an association of the presence and expression of virulence genes with biofilm production [56]. In our study, high frequencies of the virulence genes *cfb, lmb, cylE, pavA,* and *gbs67* were detected in the GBS isolates and most isolates carried multiple virulence genes, which allowed the GBS strains to adhere, colonize, and invade host tissues. However, there were no associations between virulence genes and strong biofilm producers. The *cfb*, *lmb*, *cylE,* and *pavA* genes were present in 95% of strong biofilm producers, followed by *gbs67* (81%) and *scpB* (43%), whereas *cylE* and *pavA* were present in 100% of moderate biofilm producers, followed by *cfb* (89%), *lmb* (78%), *gbs67* (67%), and *scpB* (33%). The findings suggest that the occurrence of virulence genes did not seem to be necessary for biofilm production by GBS.

As the associations of antibiotic resistance and virulence genes with biofilm formation in GBS strains had not previously been studied, this study was conducted to assess the relationships. However, no relationships were observed between antibiotic resistance and biofilm formation, except in the case of penicillin resistance, which was associated with strong biofilm formation. We recommend routine monitoring for antibiotic resistance and biofilm formation in order to determine the antibiotics that are suitable for treating GBS infections.

## Conclusion

The comprehensive study of *Streptococcus agalactiae* has not been searched into yet. The purpose of the current investigation to analyze the correlation between biofilm formation and antibiotic resistant of GBS. This study also provided the information of the virulence factors which involve in biofilm formation. Together this information might contribute as a desirable candidate for vaccine development against GBS.

Total thirty isolates were identified as GBS by their biochemical, microbiological tests and molecular test. These thirty isolates were tested against the six antibiotics which are highly utilized during the infection or disease caused by GBS. In conclusion, all antibiotics showed resistant against GBS. In fact, penicillin which is widely used as an intrapartum prophylaxis (at the time of delivery), was showing higher level of resistance (30%). Various Gram-positive bacteria have ability to generate biofilm on host anatomical sites. GBS's biofilm development can be compared to that of other biofilm communities. On performing the biofilm forming assay, it was concluded that, all thirty isolates have ability to develop biofilm. Based on their biofilm forming ability, 70% isolates were produced strong biofilm as well as 30% were formed moderate biofilm.

Biofilm communities have surplus mechanism of resistance as compared to the planktonic cells which hinder the treatment strategies and emerged antibiotic resistance. In this study, we correlated the previous results of antibiotic resistant and biofilm formation by statistical tool spearman’s correlation. It concluded that, five out of six antibiotics did not show correlation but penicillin was showing negative correlation which suggested that the acquisition of penicillin resistant was might be due to the biofilm formation.

Virulence factors play a significant influence in the disease manifestation. Therefore, the determination of relation between GBS virulence genes and biofilm has not been investigated yet. So we selected six virulence genes (*gbs67, cylE, cfb, scpB, lmb* and *pavA*) which were responsible in adhesion and invasion was amplified by conventional PCR method to analyze their presence in the isolates. Therefore, the result of this study showed that the majority of the isolates carried all the virulence genes which were functioned as invasive and colonizing. To check the association between biofilm and virulence genes, statistical tool Chi-square was performed and elucidate as the biofilm formation was not significantly associated with the expression of any virulence gene.

## Supplementary Information

**Additional file 1:** **Supplementary Table S1.** Primer sets used for the detection of virulence genes in *S. agalactiae*. **Supplementary Table S2.** Significant difference in mean value of zone of inhibition between the antibiotics. To find out the significant differences within the antibiotics, Tukey multiple comparison test was performed. **Figure S1.** Differences within the antibiotics on the basis of mean differences of the zone of inhibition. **Figure S2.** Distribution of serotypes and antibiotics resistance of *Streptococcus agalactiae* isolates. **Figure S3.** Growth rate analysis of different GBS serotypes grown in THB+1 % glucose. **Figure S4.** Biofilm formation by invasive and colonizing GBS isolates. **Supplementary Table S3.** GBS serotypes, their source of isolation, biofilm formation status, presence of virulence genes (+/-) and antibiotic susceptibility [57].

## Data Availability

All data generated or analyzed during this study are included in this published article.
