# Supplementary material for: Antibiotic resistance, biofilm formation, and virulence genes of Streptococcus agalactiae serotypes of Indian origin
Source: BMC Microbiol. 2023 Jul 5;23:176. doi: 10.1186/s12866-023-02877-y (PMC10320965; doi:10.1186/s12866-023-02877-y)
Supplement: Supplementary file 1 — Additional file 1: Supplementary Table S1. Primer sets used for the detection of virulence genes in S. agalactiae. Supplementary Table S2. Significant difference in mean value of zone of inhibition between the antibiotics. To find out the significant differences within the antibiotics, Tukey multiple comparison test was performed. Figure S1. Differences within the antibiotics on the basis of mean differences of the zone of inhibition. Figure S2. Distribution of serotypes and antibiotics resistance of Streptococcus agalactiae isolates. Figure S3. Growth rate analysis of different GBS serotypes grown in THB+1 % glucose. Figure S4. Biofilm formation by invasive and colonizing GBS isolates. Supplementary Table S3. GBS serotypes, their source of isolation, biofilm formation status, presence of virulence genes (+/-) and antibiotic susceptibility [57]. [file 12866_2023_2877_MOESM1_ESM.doc]

**Antibiotic resistance, biofilm formation and virulence genes of *Streptococcus agalactiae* serotypes of Indian origin**

Shalini Verma^1^, Monika Kumari^1^, Anurag Pathak^2^, Vikas Yadav^3^, Atul Kumar Johri^3^*, and Puja Yadav^1^*

^1^Department of Microbiology, Central University of Haryana, Mahendergarh, Haryana, India

^2^Department of Statistics, Central University of Haryana, Mahendergarh, Haryana, India

^3^School of Life Sciences, Jawaharlal Nehru University, New Delhi-110067, India

*** Correspondence:**

**Puja Yadav:** **pujayadav@cuh.ac.in**

**Atul K Johri: akjohri14@yahoo.com**

**Supplementary Table S1. Primer sets used for the detection of virulence genes in S. agalactiae**

| **Virulence genes** | **Primers (5’ to 3’)** | **Annealing temperature (^o^C)** | **References** |
| --- | --- | --- | --- |
| ***gyrA* (positive control)** | TCGTACTCAGTTTGACAAAGC  GATATATCCCTTGTTTGAGAGAG | 53 | [30] |
| ***gbs 67*** | CGAAACTGCTTCTATTAACT  GTACTTTTCAAAAATTGAAAGAG | 55 | [55] |
| ***cylE*** | TGACATTTACAAGTGACGAAG TTGCCAGGAGGAGAATAGGA | 47 | [26] |
| ***Cfb*** | ATGGGATTTGGGATAACTAAGCTAG AGCGTGTATTCCAGATTTCCTTAT | 50 | [26] |
| ***scpB*** | AGTTGCTTCTTACAGCCCAGA GGCGCAGACATACTAGTTCCA | 51 | [26] |
| ***Lmb*** | AGTCAGCAAACCCCAAACAG GCTTCCTCACCAGCTAAAACG | 50 | [26] |
| ***pavA*** | TTCCCATGATTTCAACAACAAG AACCTTTTGACCATGAATTGGTA | 47 | [26] |

**Supplementary Table S2**. Significant difference in mean value of zone of inhibition between the antibiotics. To find out the significant differences within the antibiotics, Tukey multiple comparison test was performed.

| **Antibiotics** | **Significant differences in mean value of zone of inhibition (mm)** | **P Value** |
| --- | --- | --- |
| Ofloxacin-Gentamicin | 2.8 | 0.101 |
| Clindamycin-Gentamicin | 4.4 | 0.001 |
| Erythromycin-Gentamicin | 4.6 | 0.001 |
| Azithromycin-Gentamicin | 4.6 | 0.001 |
| Penicillin-Gentamicin | 7.9 | 0.00 |
| Clindamycin-Ofloxacin | 1.5 | 0.758 |
| Erythromycin-Ofloxacin | 1.7 | 0.660 |
| Azithromycin-Ofloxacin | 1.7 | 0.628 |
| Penicillin-Ofloxacin | 5.1 | 0.001 |
| Erythromycin-Clindamycin | 0.17 | 0.9 |
| Azithromycin-Clindamycin | 0.23 | 0.9 |
| Penicillin-Clindamycin | 3.6 | 0.01 |
| Azithromycin-Erythromycin | 0.05 | 0.001 |
| Penicillin-Erythromycin | 3.4 | 0.02 |
| Penicillin- Azithromycin | 3.4 | 0.03 |


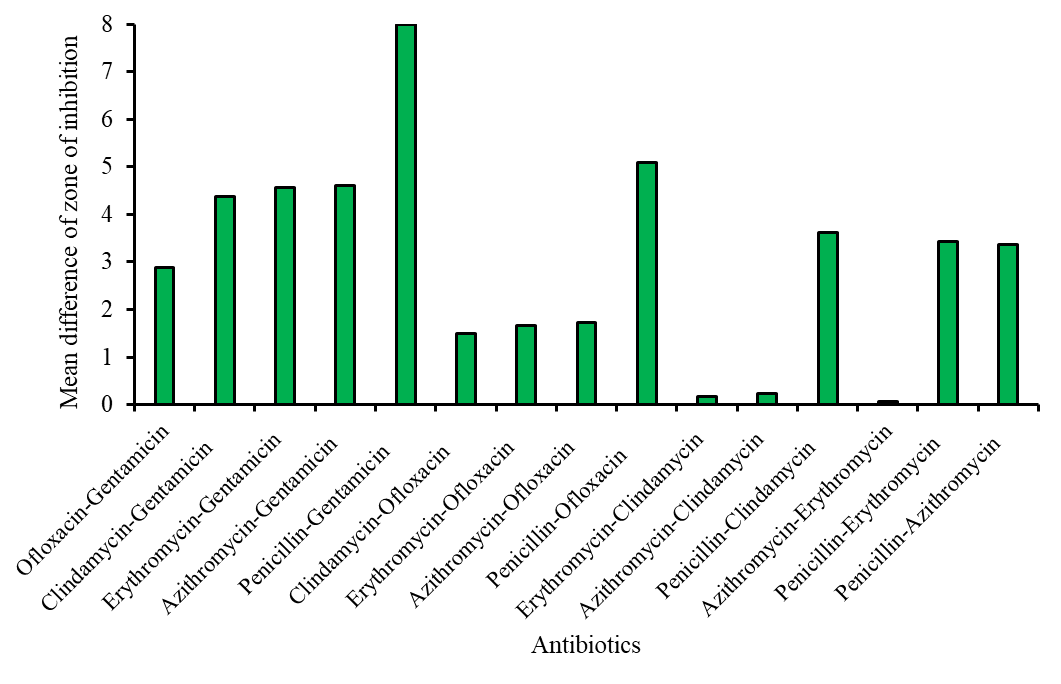


**Figure S1: Differences within the antibiotics on the basis of mean differences of the zone of inhibition.**


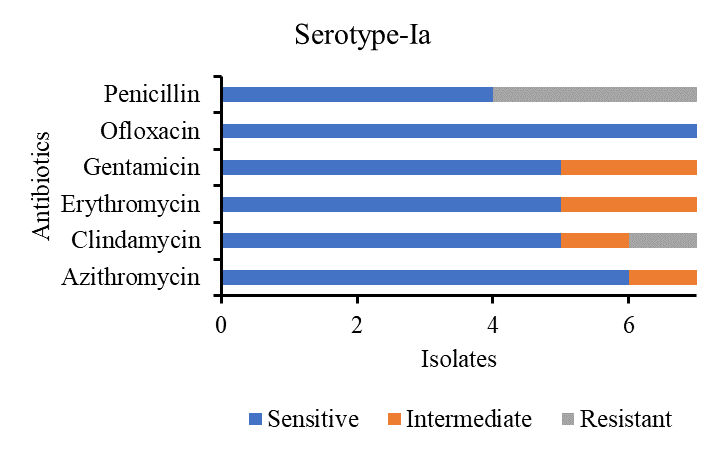

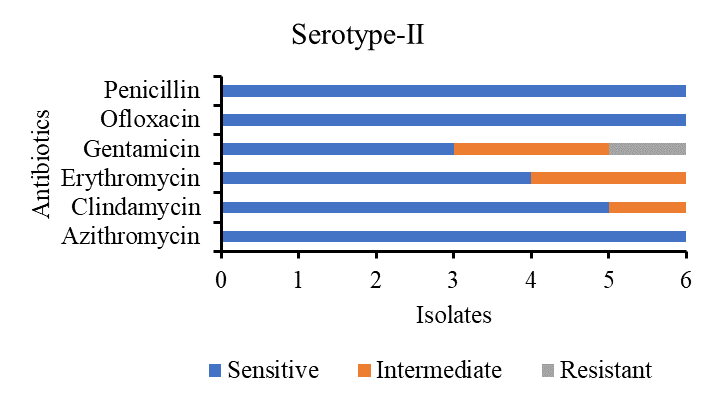

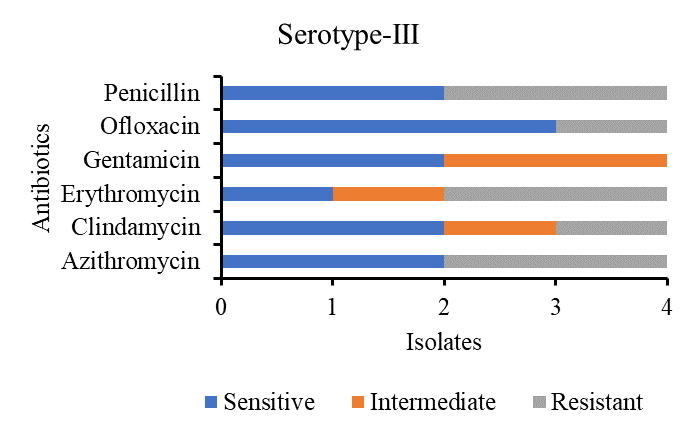

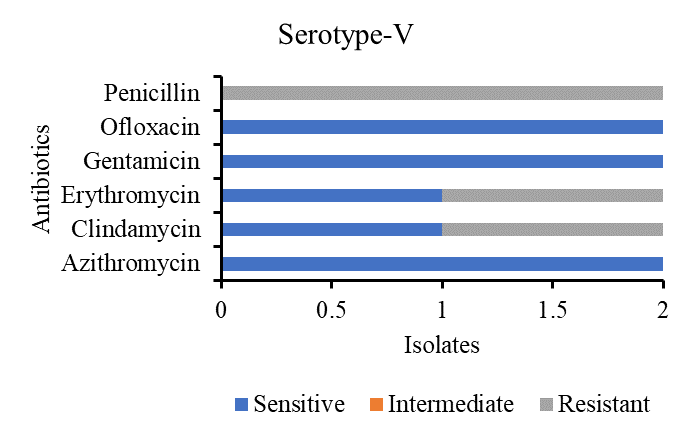

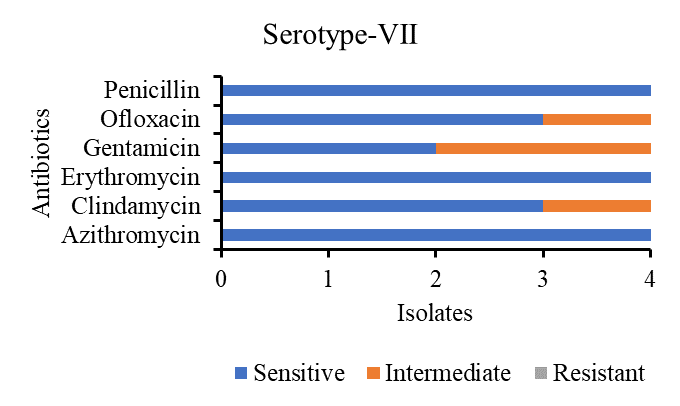


**Figure S2. Distribution of serotypes and antibiotics resistance of *Streptococcus agalactiae* isolates**

**
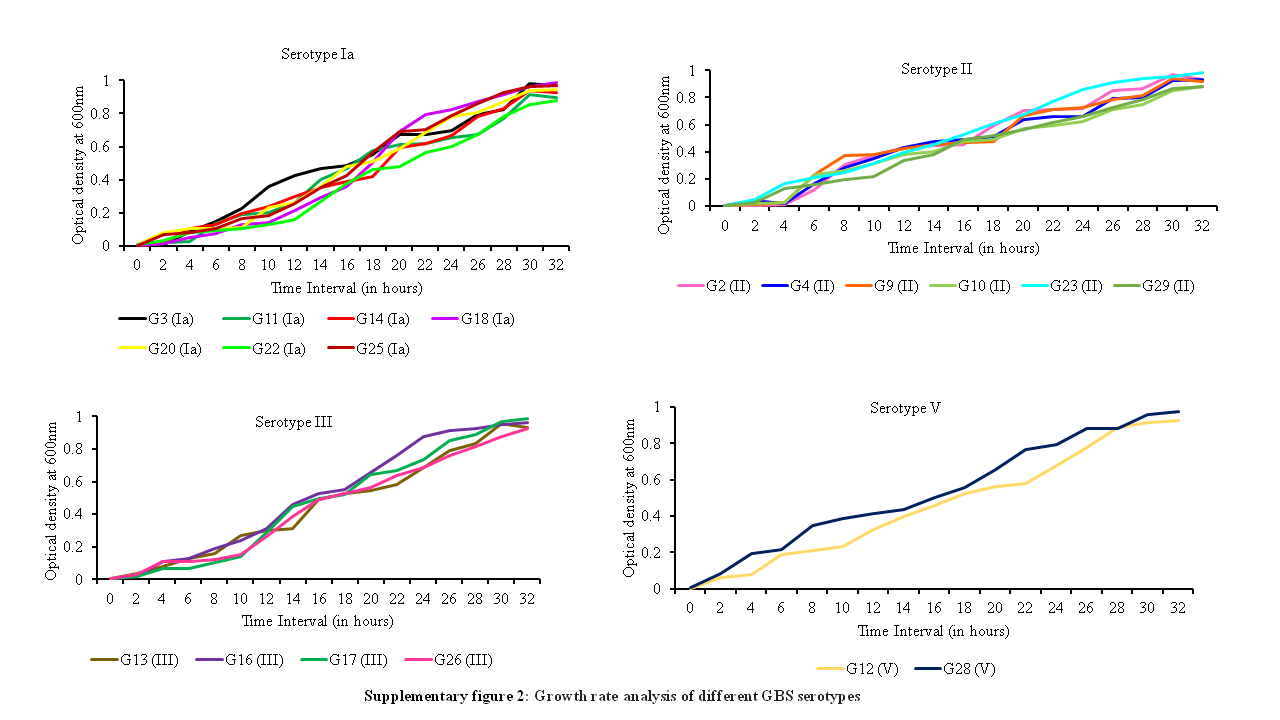

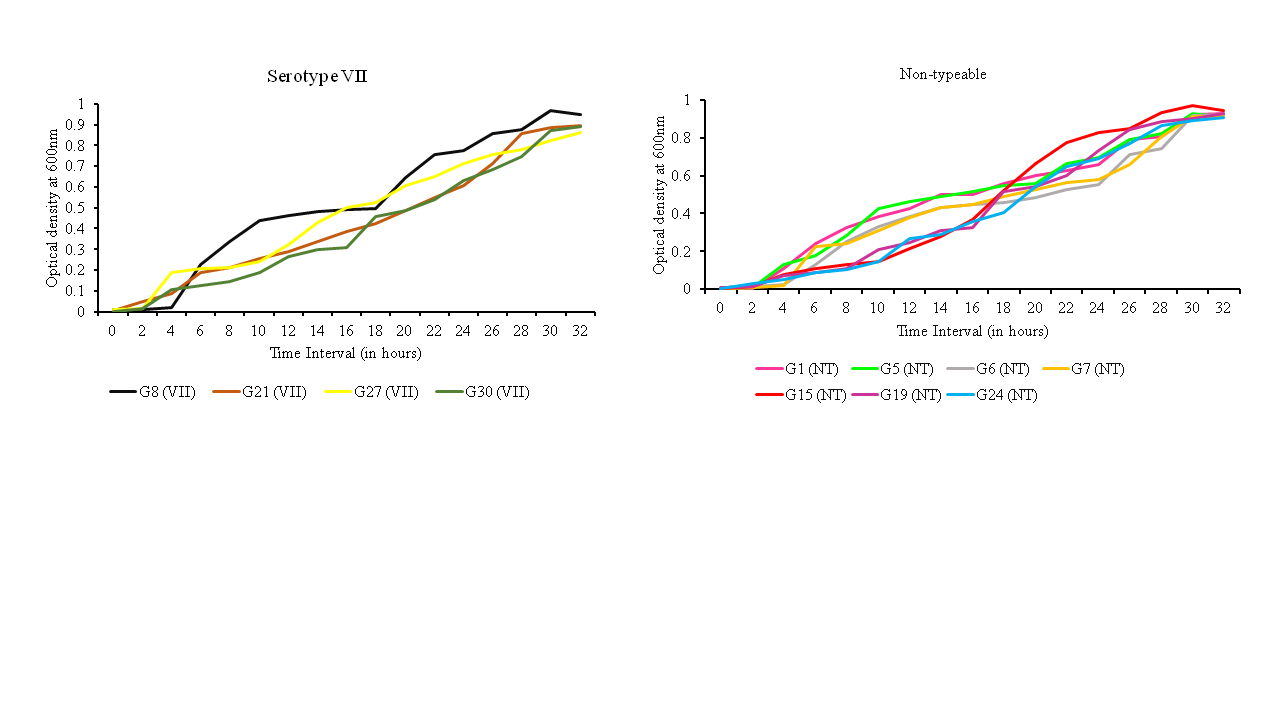
**

**Figure S3:** Growth rate analysis of different GBS serotypes grown in THB+1 % glucose.


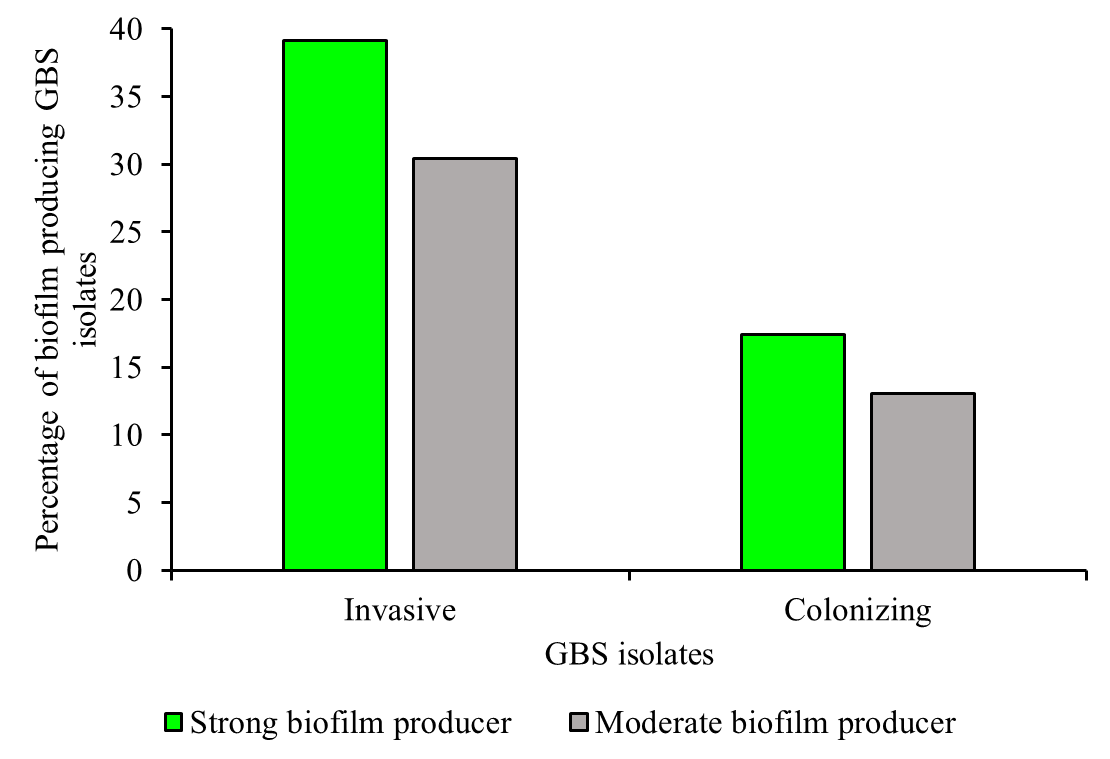


**Figure S4: Biofilm formation by invasive and colonizing GBS isolates.**

**Supplementary Table S3: GBS serotypes, their source of isolation, biofilm formation status, presence of virulence genes (+/-) and antibiotic susceptibility.**

| **Isolates (serotype)** | **Source of collection and patient details** | **Biofilm forming ability** | **Azithromycin** | **Clindamycin** | **Erythromycin** | **Gentamicin** | **Ofloxacin** | **Penicillin** | **gbs67** | **cfb** | **lmb** | **cylE** | **pavA** | **scpB** |
| --- | --- | --- | --- | --- | --- | --- | --- | --- | --- | --- | --- | --- | --- | --- |
| G1 (NT) | Urine/ no details | Strong | Sensitive | Sensitive | Sensitive | Sensitive | Sensitive | Sensitive | + | + | + | + | + | + |
| G2 (II) | Urine/ no details | Strong | Sensitive | Intermediate | Intermediate | Sensitive | Sensitive | Sensitive | - | - | + | + | + | - |
| G3 (Ia) | Urine/ no details | Strong | Sensitive | Sensitive | Sensitive | Sensitive | Sensitive | Sensitive | + | + | + | + | + | - |
| G4 (II) | Urine/ no details | Moderate | Sensitive | Sensitive | Intermediate | Intermediate | Sensitive | Sensitive | + | + | + | + | + | - |
| G5 (NT) | Urine/ 24 F | Strong | Sensitive | Sensitive | Sensitive | Sensitive | Sensitive | Sensitive | + | + | + | + | + | + |
| G6 (NT) | Urine/ 45 M | Strong | Sensitive | Sensitive | Sensitive | Sensitive | Sensitive | Sensitive | + | + | + | + | + | - |
| G7 (NT) | Urine/ 47 F | Moderate | Sensitive | Sensitive | Sensitive | Sensitive | Sensitive | Sensitive | + | - | + | + | + | + |
| G8 (VII) | Urine/ 24 F | Strong | Sensitive | Intermediate | Sensitive | Sensitive | Intermediate | Sensitive | - | + | + | + | + | + |
| G9 (II) | Urine/ 54 F | Strong | Sensitive | Sensitive | Sensitive | Sensitive | Sensitive | Sensitive | + | + | + | + | + | - |
| G10 (II) | No details/ 52 F | Strong | Sensitive | Sensitive | Sensitive | Sensitive | Sensitive | Sensitive | + | + | + | + | - | + |
| G11 (Ia) | No details/ 44 F | Strong | Sensitive | Sensitive | Sensitive | Sensitive | Sensitive | Resistant | - | + | + | + | + | - |
| G12 (V) | No details/ 50 F | Strong | Sensitive | Sensitive | Sensitive | Sensitive | Sensitive | Resistant | + | + | + | + | + | - |
| G13 (III) | Semen/ 30 M | Strong | Sensitive | Resistant | Intermediate | Sensitive | Sensitive | Resistant | + | + | + | + | + | - |
| G14 (Ia) | Urine/ M | Strong | Sensitive | Sensitive | Sensitive | Intermediate | Sensitive | Sensitive | - | + | - | + | + | - |
| G15 (NT) | Urine/ 68 M | Moderate | Sensitive | Sensitive | Sensitive | Intermediate | Sensitive | Sensitive | + | + | + | + | + | - |
| G16 (III) | Urine/ 29 F | Moderate | Resistant | Sensitive | Resistant | Intermediate | Resistant | Sensitive | + | + | + | + | + | - |
| G17 (III) | Urine/ 80 F | Moderate | Resistant | Intermediate | Resistant | Intermediate | Sensitive | Sensitive | + | + | + | + | + | - |
| G18 (Ia) | Urine/ 70 F | Strong | Intermediate | Intermediate | Intermediate | Intermediate | Sensitive | Sensitive | + | + | + | + | + | - |
| G19 (NT) | Urine/ 23 F | Strong | Sensitive | Sensitive | Sensitive | Sensitive | Sensitive | Sensitive | + | + | + | + | + | - |
| G20 (Ia) | Urine/ 83 F | Moderate | Sensitive | Sensitive | Sensitive | Sensitive | Sensitive | Sensitive | + | + | - | + | + | + |
| G21 (VII) | Urine/ 55 F | Strong | Sensitive | Sensitive | Sensitive | Sensitive | Sensitive | Sensitive | + | + | + | - | + | + |
| G22 (Ia) | Urine/ 51 F | Strong | Sensitive | Sensitive | Sensitive | Sensitive | Sensitive | Resistant | + | + | + | + | + | + |
| G23 (II) | Urine/ 56 M | Strong | Sensitive | Sensitive | Sensitive | Intermediate | Sensitive | Sensitive | + | + | + | + | + | + |
| G24 (NT) | Urine/ 26 M | Strong | Sensitive | Sensitive | Sensitive | Sensitive | Sensitive | Resistant | + | + | + | + | + | + |
| G25 (Ia) | Urine/ 13 F | Moderate | Sensitive | Resistant | Intermediate | Sensitive | Sensitive | Resistant | - | + | + | + | + | - |
| G26 (III) | Urine/ 68 F | Strong | Sensitive | Sensitive | Sensitive | Sensitive | Sensitive | Resistant | + | + | + | + | + | + |
| G27 (VII) | Urine/ 78 F | Moderate | Sensitive | Sensitive | Sensitive | Intermediate | Sensitive | Sensitive | - | + | - | + | + | + |
| G28 (V) | Urine/ 24 F | Strong | Sensitive | Resistant | Resistant | Sensitive | Sensitive | Resistant | + | + | + | + | + | - |
| G29 (II) | No details | Strong | Sensitive | Sensitive | Sensitive | Resistant | Sensitive | Sensitive | + | + | + | + | + | - |
| G30 (VII) | No details | Moderate | Sensitive | Sensitive | Sensitive | Intermediate | Sensitive | Sensitive | - | + | + | + | + | - |
